# Clinical and Communication Factors Associated With Family Conflict in Palliative Care Units: A Survey of Bereaved Families in Japan

**DOI:** 10.1002/cam4.71192

**Published:** 2025-08-30

**Authors:** Jun Hamano, Kento Masukawa, Masanori Mori, Takashi Yamaguchi, Hiroyuki Otani, Hiroto Ishiki, Yutaka Hatano, Isseki Maeda, Satoru Tsuneto, Yasuo Shima, Tatsuya Morita, Yoshiyuki Kizawa, Mitsunori Miyashita

**Affiliations:** ^1^ Department of Palliative and Supportive Care, Institute of Medicine University of Tsukuba Ibaraki Japan; ^2^ Department of Palliative Nursing, Health Sciences Tohoku University Graduate School of Medicine Sendai Miyagi Japan; ^3^ Department of Palliative and Supportive Care Seirei Mikatahara General Hospital Hamamatsu Japan; ^4^ Department of Palliative Medicine Kobe University Graduate School of Medicine Kobe Japan; ^5^ Department of Palliative Care Team, and Palliative and Supportive Care National Hospital Organization Kyushu Cancer Center Fukuoka Japan; ^6^ Department of Palliative Medicine National Cancer Center Hospital Tokyo Japan; ^7^ Department of Palliative Care Daini Kyoritsu Hospital Kawanishi Japan; ^8^ Department of Palliative Care Senri Chuo Hospital Suita Japan; ^9^ Graduate School of Medicine Kyoto University Kyoto Japan; ^10^ Hospice Palliative Care Japan Ashigarakami Kanagawa Japan; ^11^ Research Association for Community Health Hamamatsu Japan

## Abstract

**Background:**

Family conflict is a common problem in palliative care and has been identified as a potential barrier to providing appropriate care. Several demographic factors associated with family conflict have been reported; however, associated clinical factors, including symptoms, treatments, and communication, remain to be elucidated.

**Aims:**

The aim of this study was to identify symptoms, treatments, and communication factors associated with family conflict in palliative care units (PCUs).

**Methods:**

We used matched data for deceased patients from a prospective cohort study conducted between January 2017 and December 2017 of cancer patients admitted to PCUs and their bereaved families from a nationwide cross‐sectional questionnaire survey in Japan. We assessed family conflict using the Outcome‐Family Conflict Scale.

**Results:**

We sent out 667 questionnaires, of which 443 (66.4%) were returned. We excluded 81 family members who refused to participate; therefore, we analyzed 362 (81.7%) responses. The mean age of the patients who died of cancer was 73.9 ± 11.3 years, and 51.9% were men. Multivariate logistic regression revealed a significant association between rapid deterioration of the patient's condition, resulting in death within 1–2 days, and family conflict about what is meant by “a good death” (*p* = 0.044, odds ratio: 2.66), and the presence of family members who insulted or yelled at other family members (*p* = 0.005, OR: 3.22). In addition, a significant association was found between the confirmation of the family's wishes regarding CPR during hospitalization and family conflicts about healthcare decisions for their relative (*p* = 0.013, OR: 0.46), about the way a member was treating their relative (*p* = 0.014, OR: 0.43), and about what is meant by “a good death” (*p* = 0.028, OR: 0.38).

**Conclusions:**

Our findings suggest that abrupt clinical deterioration is associated with family conflict. Confirming patients' and families' wishes regarding CPR during hospitalization may help reduce family conflict in PCUs.

## 1 Introduction

End‐of‐life (EOL) care for patients with cancer is complicated by emotional, physical, and ethical challenges that often lead to family conflict [1, 2]. Family conflict has a relatively high prevalence rate of 42%, and has been identified as a potential barrier to the provision of appropriate palliative care [1, 3]. Previous studies have shown that family conflict not only negatively affects the quality of care provided to the patient but also increases caregiver burden, increases emotional distress, and leads to prolonged grief and psychological distress among the surviving family members [1, 4, 5, 6].

Families are an integral part of the decision‐making process, particularly in palliative care, where complex decisions must be made regarding resuscitation, continuation of treatment, and sedation [1, 7]. Family conflict may arise from differing opinions about these decisions, differing perceptions of the patient's condition, and unmet caregiving expectations [1, 3].

Previous studies have identified demographic factors associated with family conflict including historical family dynamics, communication limitations, lack of shared understanding of treatment goals, differing perceptions of the patient's prognosis, and cultural views of decision‐making [3, 4, 8]. However, there is a lack of knowledge about clinical factors, such as symptoms, treatments, and communication, associated with family conflict in palliative care units (PCUs).

We aimed to address the gaps in the current literature by focusing on the symptoms, treatments, and communication that contribute to family conflict during EOL care in PCUs. By identifying these factors, we hope to inform strategies that healthcare providers can implement to minimize family conflict and improve the quality of palliative care.

## 2 Methods

### 2.1 Setting and Participants

We used matched data from deceased patients and their family members. Patient data were obtained from the East Asian collaborative cross‐cultural Study to Elucidate the Dying process (EASED), a study of the dying process and EOL care of terminally ill cancer patients [9, 10].

Consecutive adult patients with advanced cancer admitted to 23 specialized PCUs in Japan were enrolled. The inclusion criteria were patients newly referred to the participating PCUs between January and December 2017. We combined the data obtained in the EASED study with those obtained in the fourth Japan Hospice and Palliative Evaluation (J‐HOPE4) study. The J‐HOPE4 study was an anonymous, cross‐sectional, self‐reported questionnaire survey of bereaved families that evaluated EOL care in Japan [11].

A self‐administered questionnaire was mailed to bereaved families between May and June 2018. The families were asked to complete and return the questionnaire within 1 month. Survivors who had been bereaved for at least 3 months were included in the study. In addition to the questionnaire, we sent a document explaining the aims and procedures of the J‐HOPE4 study. The return of a completed questionnaire was considered consent to participate. We sent a reminder to nonrespondents 1 month after sending the questionnaire. In the study, participants who did not wish to take part were asked to indicate their decision by checking a “no participation” box on the questionnaire. They were then instructed to return the incomplete questionnaire.

The J‐HOPE4 study, as well as the combined data analysis with the EASED study, received approval from the Institutional Review Board of Tohoku University Hospital (2017‐2‐236‐1) and each participating institution.

### 2.2 Participant Characteristics

#### 2.2.1 Baseline Characteristics

We asked the primary palliative care physicians to collect the demographic data (age, sex, primary tumor site, and length of admission) of each patient from their medical records. The bereaved family members were asked for demographic data such as age, sex, relationship to the patient, and education.

### 2.3 Symptoms, Received Treatments, and Communication

We identified the following 12 factors related to symptoms, treatment, and communication at admission and/or during the hospitalization period through discussions among researchers: delirium at admission, hyperactive delirium at admission, hyperactive delirium during hospitalization, inability to take oral intake for more than 1 week before death, fever within 1 week before death, infusion within 1 week before death, and deep continuous sedation, whether the patient's condition deteriorated rapidly and they died within 1–22 days, whether the patient's wish for cardiopulmonary resuscitation (CPR) was confirmed at admission, whether the patient's wish for CPR was confirmed during hospitalization, whether the family's wish for CPR was confirmed at admission, and whether the family's wish for CPR was confirmed during hospitalization.

The presence/absence of delirium was assessed at two time points (admission and during hospitalization), based on the definition in the Diagnostic and Statistical Manual of Mental Disorders, Fourth Edition [12]. If the patient developed hyperactive delirium, the severity of agitation was assessed using the Memorial Delirium Assessment Scale (MDAS) item 9 (1: mild, 2: moderate, 3: severe) [13]. Individual MDAS items are highly correlated with existing measures of delirium [14]. The presence/absence of inability to take oral intake for more than 1 week before death, fever within 1 week before death, infusion within 1 week before death, and rapid deterioration and death within 1–2 days were recorded by primary palliative care physicians.

The presence/absence of communication regarding CPR was recorded by primary palliative care physicians and assessed at two time points (admission and during hospitalization).

### 2.4 Outcome Measures

Family conflict during the patient's illness was assessed more than three months after the patient's death using the Outcome‐Family Conflict (OFC) scale. The OFC scale consists of eight items and was specifically developed to measure family conflict at the EOL [2]. This scale's construct validity has been demonstrated through significant correlations with a standardized family functioning measure, and it exhibits strong internal consistency (Cronbach's α of 0.89). Respondents answered eight questions about the EOL experience on a five‐point scale (1 = not at all, 2 = almost not, 3 = sometimes, 4 = often, 5 = very much) [2]. Higher total scores on the OFC scale correspond to higher levels of family conflict. Additionally, we calculated an “agreement” rate for each item, defined as the proportion of responses that were “sometimes,” “often,” or “very much.”

We also examined “coming out of the woodwork” in the family. This term refers to family members who had not been in regular contact with the patient but suddenly became involved as a result of the patient's illness. We measured this phenomenon with a single yes/no question (0 = no; 1 = yes) [2].

In addition, we assessed the family's level of assertion of control over patient care decisions. This measure consisted of a single question asking the extent to which any family member asserted control over decision‐making about the patient's care. Answers were recorded on a five‐point scale from 1 (not at all) to 5 (very much), with higher scores indicating a greater degree of control [2].

Communication constraints within the family were evaluated using four items from the Family Assessment Device (FAD). The FAD is a self‐report measure of perceived family functioning [15]. Participants indicated their level of agreement with the following statements on a five‐point scale (1 = strongly disagree; 5 = strongly agree) [2]: “We cannot talk to each other about the sadness we feel”; “We avoid discussing our fears and concerns”; “We can express our feelings to each other”; and “We confide in each other.” Two of these items were reverse‐coded to ensure a consistent direction of scoring, and higher overall scores reflected more severe communication constraints.

### 2.5 Statistical Analysis

We conducted a descriptive analysis for each of the eight items of the OFC score. Then, we performed a multivariable logistic regression analysis to examine the relationship between the agreement on each OFC item and the twelve factors. We defined agreement as responses of “sometimes”, “often” or “very much” for each OFC item. This analysis included adjustments for the following four variables identified by previous studies and discussion among researchers: patient age (≥ 65 years), FAD score, presence of an assertive family member, and beneficial contact with family members not previously in regular contact (trivalent variable). Significance was defined as *p* < 0.05. All analyses were performed using SPSS‐J software (ver. 27.0; IBM, Tokyo, Japan).

## 3 Results

We sent out 667 questionnaires, of which 443 (66.4%) were returned. Eighty‐one family members were excluded for refusing to participate; therefore, we analyzed a total of 362 responses (81.7%). The characteristics of the participants are summarized in Table 1. The mean age of the patients who died of cancer was 73.9 ± 11.3 years, and 51.9% were men. The most frequent primary tumor was hepatobiliary/pancreatic cancer, followed by lung cancer. The duration of hospitalization was 26.6 ± 27.0 days. Bereaved family members had a mean age of 62.1 ± 12.1 years, and 35.1% were men. The most common bereaved person was the patient's spouse (43.4%), followed by the patient's child (38.4%) (Table 1). The prevalence of the number of patients who had delirium at admission was 122 (33.7%) and those of hyperactive delirium at admission was 66 (18.2%).

**TABLE 1:** The characteristics of the participants.

	All (*n* = 362)	
	*n*	%
Patients		
Age (mean ± standard deviation)	73.9 ± 11.3	
Sex		
Male	188	51.9
Female	174	48.1
Primary tumor sites		
Liver, bile duct, pancreas	73	20.2
Lung	69	19.1
Stomach, esophagus	55	15.2
Colon, rectum	42	11.6
Prostate, kidney, bladder	27	7.5
Breast	25	6.9
Uterus, ovary	18	5.0
Head and neck, brain	14	3.9
Blood	8	2.2
Others	31	8.6
Length of admission (days, mean ± standard deviation)	26.6 ± 27.0	
Bereaved family members		
Age (mean ± standard deviation)	62.1 ± 12.1	
Sex		
Male	127	35.1
Female	227	62.7
Relationship with patients		
Husband/wife	157	43.4
Child	139	38.4
Daughter‐in‐law or son‐in‐law	10	2.8
Parents	2	0.6
Siblings	31	8.6
Others	17	4.7
Education		
Less than high school	31	8.6
High school graduate	147	40.6
Post‐high school education	172	47.5
Score of Family Assessment Device^a^ (mean ± standard deviation)	9.6 ± 3.2	
Hyperactive delirium during hospitalization	174	48.1
Inability to take oral intake for more than 1 week until death	90	24.9
Fever within 1 week before death	137	37.8
Infusion within 1 week before death	211	58.3
Deep continuous sedation	36	9.9
Patient's condition deteriorated rapidly, and they died within 1–2 days	60	
Patient's wish for cardiopulmonary resuscitation		
At the time of hospitalization	38	10.5
During hospitalization	36	9.9
Family's wish for cardiopulmonary resuscitation		
At the time of hospitalization	213	58.8
During hospitalization	221	61.0
Duration of bereavement (mean ± standard deviation, months)	14.0 ± 3.4	
Duration of bereavement (median, range, months)	14.3 (6.5–21.1)	

^a^ Higher scores indicated higher levels of communication constraints (range: 4–20).

The mean OFC score was 13.7 ± 5.1 (maximum score: 40), and 46.6% of family members reported at least one family conflict during EOL care. The most frequent family conflict was “about certain family members not pulling their weight” (25.7%), followed by “about healthcare decisions for your relative” (19.2%), “about your relative's illness or physical condition” (17.1%), and “about the way a family member was treating your relative” (17.1%) (Table 2).

**TABLE 2:** Distribution of the family conflict, physician's explanation and family relationship index.

	All (*n* = 362)	
	Mean ± SD	Agreement rate (%)
Outcome‐Family Conflict score (mean score ± standard deviation, maximum score: 40)	13.7 ± 5.1	
Outcome‐Family Conflict (mean score ± standard deviation, agreement rate^a^)		
Disagree or argue about healthcare decisions for your relative	1.9 ± 0.9	19.2
Disagree or argue about your relative's illness or physical condition	1.8 ± 0.9	17.1
Disagree or argue about the way a member was treating your relative	1.8 ± 0.9	17.1
Disagree or argue about certain family members not pulling their weight	2.0 ± 1.1	25.7
Disagree or argue about where your relative should live out his/her remaining days	1.7 ± 0.9	13.6
Disagree or argue about what is meant by “a good death”	1.6 ± 0.8	9.1
Disagree or argue about how money is being spent	1.4 ± 0.6	2.0
How much do any family members insult or yell at one another	1.5 ± 0.8	12.4
At least one family conflict		46.6
Family members who were not previously in regular contact with the patient suddenly becoming involved as a result of the patient's illness (Yes)		34.9
Any family member had asserted control in decision making regarding patient care (Yes)		5.5
Score of Family Assessment Device^b^	9.6 ± 3.2	

Abbreviation: SD, standard deviation.

^a^ Range: 1–5 (1: not at all; 5: very much), the agreement rate is the sum of “sometimes”, “often”, and “very much”.

^b^ Higher scores indicated higher levels of communication constraints (range: 4–20).

Multivariate logistic regression analysis showed a significant positive association between rapid deterioration of the patient's condition, resulting in death within 1–2 days, and family conflict about what is meant by “a good death” (*p* = 0.044, odds ratio: 2.66), and the presence of family members who insulted or yelled at other family members (*p* = 0.005, odds ratio: 3.22). In addition, the confirmation of the family's wishes regarding CPR during hospitalization was significantly negatively associated with family conflicts about healthcare decisions for their relative (*p* = 0.013, odds ratio: 0.46), about the way a member was treating their relative (*p* = 0.014, odds ratio: 0.43), and about what is meant by “a good death” (*p* = 0.028, odds ratio: 0.38) (Table 3). The area under the ROC curve for each model has shown in Data S1.

**TABLE 3:** Multivariable logistic regression analysis between each item of the OFC score and twelve factors of symptoms, treatment, and communication.

	Delirium at admission			Hyperactive delirium at admission			Hyperactive delirium during hospitalization		
	OR	95% CI	*p* value	OR	95% CI	*p* value	OR	95% CI	*p* value
Outcome‐family conflict									
Disagree or argue about healthcare decisions for your relative	1.06	0.56–2.01	0.860	0.97	0.43–2.18	0.935	1.37	0.75–2.50	0.312
Disagree or argue about your relative's illness or physical condition	1.11	0.56–2.19	0.765	1.06	0.45–2.47	0.902	1.11	0.58–2.12	0.747
Disagree or argue about the way a member was treating your relative	0.67	0.32–1.39	0.284	0.62	0.24–1.62	0.329	1.02	0.53–1.95	0.962
Disagree or argue about certain family members not pulling their weight	0.50	0.26–0.95	0.036	0.96	0.45–2.05	0.909	1.21	0.68–2.14	0.522
Disagree or argue about where your relative should live out his/her remaining days	0.89	0.38–2.07	0.782	1.72	0.67–4.42	0.258	1.48	0.67–3.28	0.338
Disagree or argue about what is meant by “a good death”	0.62	0.24–1.61	0.325	1.13	0.39–3.33	0.258	0.96	0.42–2.20	0.919
Disagree or argue about how money is being spent	0.51	0.05–4.83	0.557	0.00	n.a	0.997	0.78	0.12–5.05	0.795
How much do any family members insult or yell at one another	0.40	0.16–1.00	0.049	0.37	0.10–1.32	0.126	0.94	0.45–1.94	0.861

	Inability to take oral intake for more than 1 week until death			Fever within 1 week before death			Infusion within 1 week before death		
	OR	95% CI	*p* value	OR	95% CI	*p* value	OR	95% CI	*p* value
Outcome‐family conflict									
Disagree or argue about healthcare decisions for your relative	0.64	0.28–1.46	0.291	0.78	0.41–1.47	0.438	0.79	0.43–1.47	0.457
Disagree or argue about your relative's illness or physical condition	0.65	0.27–1.55	0.328	0.83	0.42–1.63	0.825	1.08	0.56–2.11	0.817
Disagree or argue about the way a member was treating your relative	0.56	0.22–1.39	0.208	0.85	0.43–1.67	0.638	0.66	0.34–1.29	0.228
Disagree or argue about certain family members not pulling their weight	1.49	0.72–3.07	0.282	1.69	0.94–3.04	0.083	1.49	0.82–2.70	0.196
Disagree or argue about where your relative should live out his/her remaining days	0.62	0.21–1.85	0.392	0.75	0.33–1.75	0.512	0.59	0.27–1.32	0.199
Disagree or argue about what is meant by “a good death”	0.53	0.17–1.69	0.283	0.34	0.12–0.95	0.040	0.47	0.20–1.11	0.084
Disagree or argue about how money is being spent	1.84	0.21–16.12	0.583	1.00	0.15–6.54	0.998	1.07	0.17–6.61	0.944
How much do any family members insult or yell at one another	0.82	0.32–2.10	0.674	0.44	0.19–1.02	0.056	0.63	0.30–1.34	0.230

	Continuous deep sedation			The patient's condition deteriorated rapidly and they died within 1–2 days			The patient's wish for cardiopulmonary resuscitation was confirmed at the time of admission		
	OR	95% CI	*p* value	OR	95% CI	*p* value	OR	95% CI	*p* value
Outcome‐family conflict									
Disagree or argue about healthcare decisions for your relative	0.89	0.33–2.39	0.815	1.62	0.77–3.41	0.209	0.39	0.11–1.38	0.143
Disagree or argue about your relative's illness or physical condition	0.95	0.33–2.70	0.917	1.32	0.58–3.00	0.510	0.52	0.15–1.82	0.515
Disagree or argue about the way a member was treating your relative	0.84	0.28–2.49	0.751	1.49	0.66–3.37	0.343	0.42	0.11–1.52	0.184
Disagree or argue about certain family members not pulling their weight	0.57	0.21–1.55	0.274	0.86	0.40–1.84	0.692	0.42	0.14–1.21	0.107
Disagree or argue about where your relative should live out his/her remaining days	0.81	0.22–3.04	0.754	2.25	0.91–5.53	0.079	0.14	0.01–1.35	0.089
Disagree or argue about what is meant by “a good death”	0.85	0.22–3.31	0.814	2.66	1.03–6.90	0.044	0.00	n.a	0.998
Disagree or argue about how money is being spent	0.00	n.a	0.998	5.09	0.65–39.79	0.121	0.00	n.a	0.998
How much do any family members insult or yell at one another	0.36	0.08–1.67	0.189	3.22	1.41–7.36	0.005	0.66	0.18–2.39	0.522

	The patient's wish for cardiopulmonary resuscitation was confirmed during their stay in hospital			The family's wish for cardiopulmonary resuscitation was confirmed at the time of admission			The family's wish for cardiopulmonary resuscitation was confirmed during their stay in hospital		
	OR	95% CI	*p* value	OR	95% CI	*p* value	OR	95% CI	*p* value
Outcome‐family conflict									
Disagree or argue about healthcare decisions for your relative	0.27	0.06–1.20	0.085	0.93	0.50–1.71	0.809	0.46	0.25–0.85	0.013
Disagree or argue about your relative's illness or physical condition	0.36	0.80–1.58	0.174	0.85	0.44–1.63	0.621	0.56	0.29–1.07	0.080
Disagree or argue about the way a member was treating your relative	0.13	0.02–1.05	0.056	0.56	0.29–1.08	0.085	0.43	0.22–0.84	0.014
Disagree or argue about certain family members not pulling their weight	0.65	0.24–1.77	0.395	0.56	0.32–1.00	0.050	0.65	0.36–1.16	0.150
Disagree or argue about where your relative should live out his/her remaining days	0.27	0.03–2.13	0.214	0.77	0.35–1.68	0.510	0.60	0.27–1.34	0.604
Disagree or argue about what is meant by “a good death”	0.31	0.04–2.45	0.265	0.61	0.27–1.40	0.245	0.38	0.16–0.90	0.028
Disagree or argue about how money is being spent	0.00	n.a	0.998	0.34	0.05–2.17	0.253	0.13	0.01–1.26	0.078
How much do any family members insult or yell at one another	0.75	0.21–2.75	0.667	0.75	0.36–1.56	0.446	0.57	0.27–1.19	0.134

*Note:* Analysis included adjustments with patient age (≥ 65 years), FAD score, presence of an assertive family member, and beneficial contact with family members previously not in regular contact (trivalent variable).

## 4 Discussion

To the best of our knowledge, this is the first large‐scale study to combine clinical cohort data and surveys of the bereaved family to examine the relationships between clinical practice and family conflict in EOL care for cancer patients.

The most important finding was that there was a significant positive association between the rapid deterioration of a patient's condition, leading to death within 1–2 days, and increased family conflict such as family members disagreeing over what is meant by “a good death” and members insulting or yelling at one another. This finding is consistent with previous scoping review which revealed that family disagreements over curative treatment and/or end‐of‐life care and decisions, and the dying process would contribute to family conflict [16]. This finding suggests that abrupt clinical deterioration may exacerbate existing family tensions or trigger new conflicts, possibly due to the sudden pressure and emotional distress these changes cause.

Because of recall bias in the bereaved family survey, it is not possible to state definitively whether there is a causal relationship; however, the following interpretations are possible. In the case of a patient whose condition deteriorated and who died within a short period of time, the family may have been unable to judge whether the patient had “a good death”, which led to conflict within the family. It is also possible that the family's regret that the patient may not have had “a good death” led to the emergence of family members who disagreed with the family's decisions and ideas, which may have led to conflict. With this in mind, when a patient deteriorates and dies within a short period of time, it is useful to assess whether there are differences in the family's understanding of the process of EOL care and, if there are differences in understanding, to communicate that as healthcare professionals, we understand that families may have different concerns, but we believe that the best possible care was provided for the patient.

The second important finding was that when there was clear communication between the family and the physician about the family's wishes regarding CPR, there was less family conflict. This suggests that effective communication and consensus building between the physician and the family regarding future medical treatment play an important role in reducing family conflict. Our study found that CPR discussions were more frequent compared with a previous study that examined the relationship between EOL discussions and family mental health in three East Asian countries [10]. This may be related to our study being limited to PCUs, whereas the previous study included bereaved families in PCUs, PCTs, and home care. In PCUs, where the palliative care physician serves as the attending physician, there is typically more frequent and structured communication aimed at reaching agreement on future medical treatment decisions such as the implementation of CPR.

We found no significant association between continuous deep sedation and family conflict. This finding supports a previous study that reported that continuous deep sedation was not associated with the development of depression in bereaved families [17]. A possible interpretation is that the majority of the families were satisfied with the outcome of sedation and the timing of sedation initiation. Another interpretation is that the family perceived continuous deep sedation as a typical palliative care option. This supports a previous study by Jonker et al., which reported that shared decision‐making among the patient, family, and healthcare providers regarding the initiation of continuous deep sedation has contributed to its establishment as a standard palliative care option [18].

## 5 Limitations

This study is subject to several limitations. First, given the study's design, we were unable to eliminate the possibility of recall bias. However, several previous studies conducted 3–12 months after a patient's death have suggested that this interval may be reasonable for limiting recall bias while also respecting the grieving process [19, 20, 21, 22, 23]. Second, although our study did not detect an association between past and present family conflict, prior studies have reported a significant correlation between these factors [1, 2]. Nonetheless, this limitation does not necessarily undermine the credibility of our findings. Third, our results may have been influenced by Japanese culture, which is traditionally family‐centered. This cultural context could lead to differences in factors such as the prevalence of family conflict when compared with other cultural settings. Therefore, caution should be exercised in generalizing our findings to other cultures. Fourth, we excluded severely distressed family members from participation. This decision may have introduced sampling bias and led to an underestimation of the prevalence of family conflict. Although conducting a bereavement survey among family members in severe distress is ethically challenging, this potential sampling bias should be taken into account when interpreting our results. Fifth, although the response rate was moderate, the participants in this study were limited to bereaved families of patients who died in 23 PCUs in Japan. As a result, selection bias may have influenced our results, and they may not be generalizable worldwide. Sixth, we did not adjust for multiple testing. Therefore, the significance of some results may have been overestimated. Seventh, while the reliability of the OFC score as a whole has been evaluated, the reliability and validity of our item‐by‐item analysis remain unconfirmed. Nonetheless, we chose to undertake this item‐level analysis because we hypothesized that the individual items of the OFC score related to symptoms and treatment could differ.

## 6 Clinical Implications

Our findings suggest that healthcare providers should focus on improving communication within families and between families and healthcare teams. This includes encouraging early discussions about treatment options and expected outcomes to avoid misunderstandings and conflicts. Providing counseling and support services for families can also reduce emotional distress and improve relationships during these difficult times. In addition, incorporating systematic family involvement practices into palliative care protocols can greatly benefit the emotional well‐being of patients and their families, leading to more effective EOL care.

## 7 Conclusion

Our results suggest that abrupt clinical deterioration is associated with family conflict. Moreover, confirming patients' and their families' wishes regarding CPR during hospitalization may help reduce family conflict in PCUs.

## Author Contributions

**Jun Hamano:** conceptualization (equal), data curation (equal), formal analysis (equal), funding acquisition (equal), investigation (equal), methodology (equal), project administration (equal), writing – original draft (equal), writing – review and editing (equal). **Kento Masukawa:** conceptualization (equal), data curation (equal), formal analysis (equal), funding acquisition (equal), investigation (equal), methodology (equal), project administration (equal), writing – review and editing (equal). **Masanori Mori:** conceptualization (equal), data curation (equal), formal analysis (equal), funding acquisition (equal), investigation (equal), methodology (equal), project administration (equal), writing – review and editing (equal). **Takashi Yamaguchi:** conceptualization (equal), data curation (equal), formal analysis (equal), funding acquisition (equal), investigation (equal), methodology (equal), project administration (equal), writing – review and editing (equal). **Hiroyuki Otani:** conceptualization (equal), data curation (equal), formal analysis (equal), funding acquisition (equal), investigation (equal), methodology (equal), project administration (equal), writing – review and editing (equal). **Hiroto Ishiki:** conceptualization (equal), data curation (equal), formal analysis (equal), funding acquisition (equal), investigation (equal), methodology (equal), project administration (equal), writing – review and editing (equal). **Yutaka Hatano:** conceptualization (equal), data curation (equal), formal analysis (equal), funding acquisition (equal), investigation (equal), methodology (equal), project administration (equal), writing – review and editing (equal). **Isseki Maeda:** conceptualization (equal), data curation (equal), formal analysis (equal), funding acquisition (equal), investigation (equal), methodology (equal), project administration (equal), writing – review and editing (equal). **Satoru Tsuneto:** conceptualization (equal), data curation (equal), formal analysis (equal), funding acquisition (equal), investigation (equal), methodology (equal), project administration (equal), writing – review and editing (equal). **Yasuo Shima:** conceptualization (equal), data curation (equal), formal analysis (equal), funding acquisition (equal), investigation (equal), methodology (equal), project administration (equal), writing – review and editing (equal). **Tatsuya Morita:** conceptualization (equal), data curation (equal), formal analysis (equal), funding acquisition (equal), investigation (equal), methodology (equal), project administration (equal), writing – review and editing (equal). **Yoshiyuki Kizawa:** conceptualization (equal), data curation (equal), formal analysis (equal), funding acquisition (equal), investigation (equal), methodology (equal), project administration (equal), writing – review and editing (equal). **Mitsunori Miyashita:** conceptualization (equal), data curation (equal), formal analysis (equal), funding acquisition (equal), investigation (equal), methodology (equal), project administration (equal), writing – review and editing (equal).

## Conflicts of Interest

The authors declare no conflicts of interest.

## Supporting information


**Data S1:** Area under the ROC Curve of multivariable logistic regression models.

## Data Availability

The data that support the findings of this study are available from the corresponding author upon reasonable request.
