# Correction to Clinical and Communication Factors Associated With Family Conflict in Palliative Care Units: A Survey of Bereaved Families in Japan

**DOI:** 10.1002/cam4.71310

**Published:** 2025-10-22

Hamano J , Masukawa K , Mori M , Yamaguchi T. , Otani H. , Ishiki H. , Hatano Y. , Maeda I. , Tsuneto S. , Shima Y. , Morita T. , Kizawa Y. , Miyashita M. Clinical and Communication Factors Associated With Family Conflict in Palliative Care Units: A Survey of Bereaved Families in Japan, Cancer Med 14:17 (2025): e71192, 10.1002/cam4.71192.40884162 PMC12397686

In the Funding section, the grant number should be 23EA1021.

We apologize for this error.

