# Supplementary material for: Clinical and Communication Factors Associated With Family Conflict in Palliative Care Units: A Survey of Bereaved Families in Japan
Source: Cancer Med. 2025 Aug 30;14(17):e71192. doi: 10.1002/cam4.71192 (PMC12397686; doi:10.1002/cam4.71192)
Supplement: Supplementary file 1 — Data S1: Area under the ROC Curve of multivariable logistic regression models. [file CAM4-14-e71192-s001.docx]

Supplement 1 Area Under the ROC Curve of multivariable logistic regression models

|  | Delirium at admission | | | | Hyperactive delirium at admission | | | | Hyperactive delirium during hospitalization | | | |
| --- | --- | --- | --- | --- | --- | --- | --- | --- | --- | --- | --- | --- |
|  | Area Under the ROC Curve | p value | 95% CI | | Area Under the ROC Curve | p value | 95% CI | | Area Under the ROC Curve | p value | 95% CI | |
| Outcome-Family Conflict |  |  |  |  |  |  |  |  |  |  |  |  |
| Disagree or argue about health care decisions for your relative | 0.620 | 0.007 | 0.532 | 0.709 | 0.617 | 0.009 | 0.529 | 0.705 | 0.637 | 0.001 | 0.557 | 0.717 |
| Disagree or argue about your relative’s illness or physical condition | 0.621 | 0.016 | 0.522 | 0.719 | 0.617 | 0.018 | 0.520 | 0.713 | 0.612 | 0.023 | 0.515 | 0.710 |
| Disagree or argue about the way a member was treating your relative | 0.708 | <0.001 | 0.622 | 0.794 | 0.708 | <0.001 | 0.621 | 0.794 | 0.689 | <0.001 | 0.599 | 0.779 |
| Disagree or argue about certain family members not pulling their weight | 0.734 | <0.001 | 0.668 | 0.800 | 0.717 | <0.001 | 0.649 | 0.786 | 0.718 | <0.001 | 0.648 | 0.787 |
| Disagree or argue about where your relative should live out his/her remaining days | 0.752 | <0.001 | 0.664 | 0.840 | 0.755 | <0.001 | 0.671 | 0.839 | 0.768 | <0.001 | 0.677 | 0.859 |
| Disagree or argue about what is meant by "a good death" | 0.740 | <0.001 | 0.633 | 0.848 | 0.734 | <0.001 | 0.637 | 0.832 | 0.728 | <0.001 | 0.628 | 0.828 |
| Disagree or argue about how money is being spent | 0.805 | 0.002 | 0.611 | 1.000 | 0.807 | 0.003 | 0.607 | 1.007 | 0.743 | 0.057 | 0.493 | 0.993 |
| How much do any family members insult or yell at one another | 0.754 | <0.001 | 0.668 | 0.839 | 0.753 | <0.001 | 0.671 | 0.835 | 0.733 | <0.001 | 0.647 | 0.819 |

|  | Inability to take oral intake for more than one week until death | | | | Fever within one week before death | | | | Infusion within one week before death | | | |
| --- | --- | --- | --- | --- | --- | --- | --- | --- | --- | --- | --- | --- |
|  | Area Under the ROC Curve | p value | 95% CI | | Area Under the ROC Curve | p value | 95% CI | | Area Under the ROC Curve | p value | 95% CI | |
| Outcome-Family Conflict |  |  |  |  |  |  |  |  |  |  |  |  |
| Disagree or argue about health care decisions for your relative | 0.618 | 0.021 | 0.518 | 0.718 | 0.614 | 0.008 | 0.530 | 0.698 | 0.632 | 0.003 | 0.545 | 0.720 |
| Disagree or argue about your relative’s illness or physical condition | 0.658 | 0.004 | 0.551 | 0.765 | 0.610 | 0.021 | 0.516 | 0.703 | 0.613 | 0.023 | 0.516 | 0.711 |
| Disagree or argue about the way a member was treating your relative | 0.690 | <0.001 | 0.586 | 0.795 | 0.689 | <0.001 | 0.600 | 0.778 | 0.710 | <0.001 | 0.625 | 0.794 |
| Disagree or argue about certain family members not pulling their weight | 0.730 | <0.001 | 0.647 | 0.813 | 0.724 | <0.001 | 0.659 | 0.790 | 0.724 | <0.001 | 0.656 | 0.792 |
| Disagree or argue about where your relative should live out his/her remaining days | 0.797 | <0.001 | 0.707 | 0.887 | 0.758 | <0.001 | 0.670 | 0.846 | 0.768 | <0.001 | 0.684 | 0.852 |
| Disagree or argue about what is meant by "a good death" | 0.761 | <0.001 | 0.652 | 0.870 | 0.753 | <0.001 | 0.663 | 0.842 | 0.745 | <0.001 | 0.650 | 0.840 |
| Disagree or argue about how money is being spent | 0.768 | <0.001 | 0.623 | 0.914 | 0.741 | 0.052 | 0.498 | 0.984 | 0.759 | 0.041 | 0.510 | 1.008 |
| How much do any family members insult or yell at one another | 0.761 | <0.001 | 0.671 | 0.852 | 0.757 | <0.001 | 0.677 | 0.838 | 0.754 | <0.001 | 0.671 | 0.837 |

|  | Continuous deep sedation | | | | The patient's condition deteriorated rapidly and they died within 1-2 days | | | | The patient's wish for cardiopulmonary resuscitation was confirmed at the time of admission | | | |
| --- | --- | --- | --- | --- | --- | --- | --- | --- | --- | --- | --- | --- |
|  | Area Under the ROC Curve | p value | 95% CI | | Area Under the ROC Curve | p value | 95% CI | | Area Under the ROC Curve | p value | 95% CI | |
| Outcome-Family Conflict |  |  |  |  |  |  |  |  |  |  |  |  |
| Disagree or argue about health care decisions for your relative | 0.612 | 0.013 | 0.523 | 0.700 | 0.623 | 0.006 | 0.536 | 0.710 | 0.641 | 0.001 | 0.556 | 0.726 |
| Disagree or argue about your relative’s illness or physical condition | 0.605 | 0.036 | 0.507 | 0.704 | 0.615 | 0.019 | 0.519 | 0.711 | 0.633 | 0.006 | 0.538 | 0.729 |
| Disagree or argue about the way a member was treating your relative | 0.691 | <0.001 | 0.600 | 0.781 | 0.698 | <0.001 | 0.611 | 0.785 | 0.699 | <0.001 | 0.611 | 0.787 |
| Disagree or argue about certain family members not pulling their weight | 0.717 | <0.001 | 0.647 | 0.788 | 0.712 | <0.001 | 0.643 | 0.782 | 0.726 | <0.001 | 0.657 | 0.794 |
| Disagree or argue about where your relative should live out his/her remaining days | 0.748 | <0.001 | 0.657 | 0.839 | 0.769 | <0.001 | 0.677 | 0.860 | 0.775 | <0.001 | 0.696 | 0.854 |
| Disagree or argue about what is meant by "a good death" | 0.727 | <0.001 | 0.622 | 0.833 | 0.739 | <0.001 | 0.632 | 0.847 | 0.771 | <0.001 | 0.682 | 0.859 |
| Disagree or argue about how money is being spent | 0.776 | 0.010 | 0.567 | 0.984 | 0.820 | 0.004 | 0.603 | 1.036 | 0.801 | 0.004 | 0.595 | 1.006 |
| How much do any family members insult or yell at one another | 0.740 | <0.001 | 0.651 | 0.829 | 0.752 | <0.001 | 0.662 | 0.842 | 0.744 | <0.001 | 0.661 | 0.827 |

|  | The patient's wish for cardiopulmonary resuscitation was confirmed during their stay in hospital | | | | The family's wish for cardiopulmonary resuscitation was confirmed at the time of admission | | | | The family's wish for cardiopulmonary resuscitation was confirmed during their stay in hospital | | | |
| --- | --- | --- | --- | --- | --- | --- | --- | --- | --- | --- | --- | --- |
|  | Area Under the ROC Curve | p value | 95% CI | | Area Under the ROC Curve | p value | 95% CI | | Area Under the ROC Curve | p value | 95% CI | |
| Outcome-Family Conflict |  |  |  |  |  |  |  |  |  |  |  |  |
| Disagree or argue about health care decisions for your relative | 0.643 | 0.001 | 0.559 | 0.726 | 0.618 | 0.009 | 0.529 | 0.707 | 0.673 | <0.001 | 0.587 | 0.758 |
| Disagree or argue about your relative’s illness or physical condition | 0.630 | 0.006 | 0.537 | 0.724 | 0.624 | 0.010 | 0.530 | 0.718 | 0.652 | 0.001 | 0.566 | 0.739 |
| Disagree or argue about the way a member was treating your relative | 0.725 | <0.001 | 0.641 | 0.808 | 0.709 | <0.001 | 0.623 | 0.795 | 0.715 | <0.001 | 0.630 | 0.799 |
| Disagree or argue about certain family members not pulling their weight | 0.715 | <0.001 | 0.646 | 0.785 | 0.732 | <0.001 | 0.665 | 0.799 | 0.719 | <0.001 | 0.651 | 0.788 |
| Disagree or argue about where your relative should live out his/her remaining days | 0.762 | <0.001 | 0.676 | 0.849 | 0.756 | <0.001 | 0.670 | 0.841 | 0.757 | <0.001 | 0.670 | 0.844 |
| Disagree or argue about what is meant by "a good death" | 0.727 | <0.001 | 0.628 | 0.826 | 0.750 | <0.001 | 0.651 | 0.848 | 0.752 | <0.001 | 0.652 | 0.852 |
| Disagree or argue about how money is being spent | 0.763 | 0.022 | 0.539 | 0.988 | 0.796 | 0.016 | 0.556 | 1.036 | 0.785 | 0.035 | 0.520 | 1.050 |
| How much do any family members insult or yell at one another | 0.736 | <0.001 | 0.649 | 0.822 | 0.741 | <0.001 | 0.656 | 0.827 | 0.748 | <0.001 | 0.662 | 0.834 |
